# Magnesium Sulphate in Urinary Retention

**Published:** 1914-12

**Authors:** 


**Magnesium Sulphate in Urinary Retention.** Waitachevsky, "Vratcheb Gaz.," February 16, 1914, reports a case of a man aged 29, complete urinary retention, due to fall from horse five years previously, no mechanic obstruction, permanent catheter not tolerated. Treated by daily injections of 1 c. c. of 25% magnesium sulphate, increased to 1½ and 2 c.c. Total of 15 injections, produced spontaneous urination. Treatment repeated after three months. Apparent permanent cure.

